# Evolution of electron transfer out of the cell: comparative genomics of six *Geobacter *genomes

**DOI:** 10.1186/1471-2164-11-40

**Published:** 2010-01-17

**Authors:** Jessica E Butler, Nelson D Young, Derek R Lovley

**Affiliations:** 1 Department of Microbiology, University of Massachusetts, Amherst, MA, USA

## Abstract

**Background:**

*Geobacter *species grow by transferring electrons out of the cell - either to Fe(III)-oxides or to man-made substances like energy-harvesting electrodes. Study of *Geobacter sulfurreducens *has shown that TCA cycle enzymes, inner-membrane respiratory enzymes, and periplasmic and outer-membrane cytochromes are required. Here we present comparative analysis of six *Geobacter *genomes, including species from the clade that predominates in the subsurface. Conservation of proteins across the genomes was determined to better understand the evolution of *Geobacter *species and to create a metabolic model applicable to subsurface environments.

**Results:**

The results showed that enzymes for acetate transport and oxidation, and for proton transport across the inner membrane were well conserved. An NADH dehydrogenase, the ATP synthase, and several TCA cycle enzymes were among the best conserved in the genomes. However, most of the cytochromes required for Fe(III)-reduction were not, including many of the outer-membrane cytochromes. While conservation of cytochromes was poor, an abundance and diversity of cytochromes were found in every genome, with duplications apparent in several species.

**Conclusions:**

These results indicate there is a common pathway for acetate oxidation and energy generation across the family and in the last common ancestor. They also suggest that while cytochromes are important for extracellular electron transport, the path of electrons across the periplasm and outer membrane is variable. This combination of abundant cytochromes with weak sequence conservation suggests they may not be specific terminal reductases, but rather may be important in their heme-bearing capacity, as sinks for electrons between the inner-membrane electron transport chain and the extracellular acceptor.

## Background

Species of the *Geobacter *clade specialize in the oxidation of organic compounds to carbon dioxide coupled to the reduction of insoluble, extracellular electron acceptors [1]. These species play an important role in pristine sediments and soils where they oxidize fermentation by-products like acetate and reduce naturally occurring insoluble Fe(III) and Mn(IV) oxides [1]. In addition, they play important roles in three biotechnical applications: they are able to degrade hydrocarbon contaminants in soils, they are able to insolubilize uranium in contaminated aquifers, and finally, they are able to transfer electrons from a variety of substrates onto graphite electrodes, from which electricity can be harvested [2-4].

The mechanisms of electron transfer to Fe(III) and extracellular electron acceptors generally are not well understood [1]. While soluble electron acceptors like oxygen and nitrate can diffuse into the cell, *Geobacter *species must transfer electrons onto an essentially insoluble, and therefore extracellular, electron acceptor. *Geobacter sulfurreducens *is currently the model organism for the *Geobacteraceae *family; the genome is sequenced [5] and there is a genetic system [6]. *G. sulfurreducens *completely oxidizes the electron donor acetate to carbon dioxide via TCA cycle reactions [7]. Electrons are then transferred into the inner membrane, presumably via NADH dehydrogenase(s) [8], and a succinate dehydrogenase [9]. Electron transfer out of the inner membrane, through the periplasm and outer membrane to Fe(III) presumably requires *c*-type cytochromes. Several cytochromes have been shown to be required for growth by Fe(III) reduction, both in *G. sulfurreducens *[10-16] and in the other well-studied dissimilatory Fe(III) reducer, *Shewanella oneidensis *[17,18]. However, a specific electron transport chain to extracellular Fe(III) has not been determined for any organism.

The genomes of several closely related Fe(III)-reducing organisms in the *Geobacter *family have recently been sequenced. This work compares the complete or 10×-coverage draft genome sequences of six species: *G. sulfurreducens, Geobacter metallireducens, Geobacter uraniireducens, Geobacter bemidjiensis, Geobacter strain FRC-32 *and *Geobacter lovleyi*. The six *Geobacter *genomes were compared and conservation of electron transport proteins was determined in order to identify electron transport genes that may be critical for the reduction of Fe(III) and other terminal electron acceptors, to better understand the evolution of the family, and to help provide foundational data for modeling of subsurface bioremediation.

## Results and Discussion

### Identification of the protein families in the six *Geobacter *genomes

The general features of each of the six genomes are presented in Table 1. Orthologous proteins, those proteins predicted to have similar functions in the different species, were identified by Markov clustering of sets of reciprocal best BLAST matches [19]. Using all 22,434 protein coding genes in the six genomes (see Additional file 1), 4,062 protein families with at least two orthologs were defined (see Additional file 2). The families contained 17,620 (79%) of all proteins. 4815 proteins were found in only one genome, and 2,196 of these were considered to be from lateral gene transfer (see Additional file 1) (discussed below). A functional role was associated with each family using the *G. sulfurreducens in silico *model annotation [20] and COG categorization [21].

**Table 1:** Characteristics of genomes used in the comparative analysis

	***Geobacter bemidjiensis***	***Geobacter sp. FRC-32***	***Geobacter lovleyi***	***Geobacter metallireducens***	***Geobacter sulfurreducens***	***Geobacter uraniireducens***
	
**NCBI ID**	NC_011146	NZ_AASH00000000	NC_010814	NC_007517	NC_002939	NC_009483
**Contigs**	1	164	1	1	1	1
**Length (nt)**	4,615,150	3,982,463	3,917,761	3,997,420	3,814,139	5,136,364
**GC Content (%)**	60	53	54	59	60	54
**Protein coding**	4018	3434	3606	3519	3446	4357
**rRNA operons**	4	1	2	2	2	2
**Plasmids**	none	n/a	77 kb	13.8 kb	none	none

For each protein family, its phyletic pattern - the pattern of which species encode the proteins in that family - was determined (see Additional files 2 and 3). By far the most common pattern was conservation across all species, 35% of the proteins (7,774) were in families that included at least one ortholog from each genome (see Additional file 3). The second most common pattern was conservation in all of the species except *G. lovleyi *- 6% (1,246) of the proteins had this phyletic pattern (see Additional file 3).

Forty-three protein families had at least 10 members (see Additional file 2). Thirteen of these large families were putative transposases which can be expected to be present in many copies in a genome. Other large protein families included three cytochrome families (protein family IDs 23, 31, and 45), a nickel-dependent hydrogenase family (ID 41), and two histidine kinase sensor/regulator families (IDs 7, 39) (Table 2).

**Table 2:** The largest families of orthologous proteins (at least 10 members) excluding transposases

ID	total members	Phyletic pattern	Function
6	16	GsGmGuGfGbGl	ATP-dependent protease La
8	16	GsGmGuGfGbGl	elongation factor G
9	16	GsGmGuGfGbGl	acetyl-CoA hydrolase/transferase
7	15	GsGmGuGfGbGl	sensory box histidine kinase
11	14	GsGmGuGfGbGl	CzcA family heavy metal efflux protein
14	14	GsGmGuGfGbGl	glycosyl transferase, group 1
15	14	GsGmGuGfGbGl	sodium/solute symporter family protein
16	13	Gs--GuGf--Gl	group II intron, maturase
17	12	--GmGu------	hypothetical protein
18	12	--Gm--Gf--Gl	Fis family transcriptional regulator
19	12	GsGmGuGfGbGl	iron-sulfur cluster-binding protein
20	12	GsGmGuGfGbGl	electron transfer flavoprotein, alpha subunit
21	12	GsGmGuGfGbGl	molybdenum cofactor biosynthesis protein A
22	12	GsGmGuGfGbGl	1-deoxy-D-xylulose-5-phosphate synthase
23	12	GsGmGuGfGb--	cytochrome c family protein
24	12	GsGmGuGfGbGl	Hybrid cluster protein
25	12	GsGmGuGfGbGl	potassium transporter family protein
30	11	GsGmGuGfGbGl	cold-shock domain-contain protein
31	11	GsGmGuGfGb--	high-molecular-weight cytochrome c
32	11	GsGmGuGfGbGl	elongation factor Tu
27	10	----Gu------	hypothetical protein
33	10	----GuGfGb--	ATPase-like
36	10	GsGmGuGfGb--	methyl-accepting chemotaxis protein
38	10	GsGmGuGfGbGl	glycogen phosphorylase
39	10	GsGmGuGfGbGl	sensor histidine kinase/response regulator
40	10	GsGmGuGfGbGl	DNA-binding response regulator
41	10	GsGmGu--GbGl	nickel-dependent hydrogenase, large subunit
42	10	GsGmGuGfGbGl	malic enzyme
43	10	GsGmGuGfGbGl	hypothetical protein GSU3410
45	10	GsGmGuGfGbGl	cytochrome c family protein

### Phylogenetics

**Figure 1:**
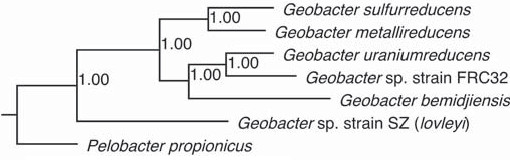
**Genome-based *Geobacter *phylogeny**. Bayesian inference of the phylogenetic tree of the six *Geobacter *species discussed, using another *Geobacteraceae *species, *Pelobacter propionicus*, as the outgroup. The tree was based on a concatenation of the proteins in the 697 families that had exactly one ortholog conserved in each of the seven genomes (listed in Additional file 4 of the supplementary material). Values at branch points are posterior probabilities.

A phylogeny of the family was constructed using the 697 protein families that had a single ortholog in each of the six genomes and the outgroup species *Pelobacter propionicus *(see Additional file 4). These proteins from each genome were concatenated then aligned, and this alignment was used to create a Bayseian model of the phylogeny (Figure 1). These proteins included many in addition to the housekeeping genes classically used to determine phylogeny, including proteins involved in information storage, metabolism, cell signaling, and those with no known function (see Additional file 4). The resulting phylogeny supports 16S rDNA phylogeny [22], and shows that the subsurface species from the contaminated bioremediation sites, *G. uraniireducens, G. *strain FRC-32, and *G. bemidjiensis *form a group distinct from the model organisms (Figure 1). Analysis of the clustering of proteins into families showed that relatively few protein families (89, made up of 283 proteins) were found only in the three subsurface species, including a hydrogenase discussed below (see Additional file 5).

### Conservation of acetate and hydrogen metabolism

**Figure 2:**
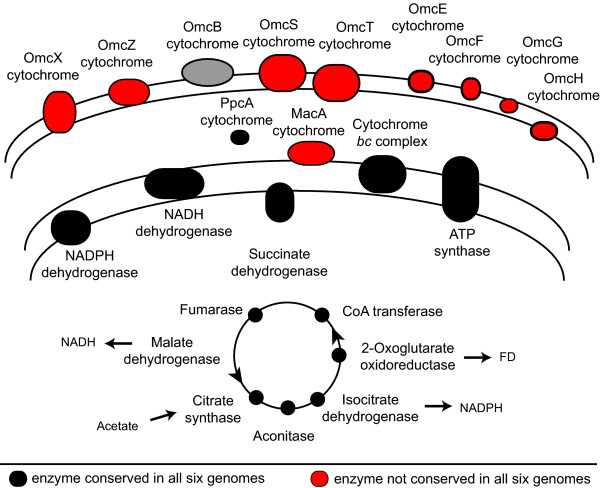
**Conservation of the energy metabolism pathways of *Geobacter sulfurreducens***. Shown are the pathways for acetate activation and oxidation via the TCA cycle in the cytoplasm; inner membrane oxidation of TCA cycle products coupled with electron/proton transport and ATP generation; and periplasmic and outer membrane cytochromes known to be required *in vivo *for transfer of electrons to an extracellular acceptor. The genes encoding the enzymes of these pathways and their full conservation pattern across all of the *Geobacter *genomes are listed in Additional file 6 of the supplementary material. The enzymes are colored black if there were orthologs for every subunit in all of the species and red if there were not. OmcB is shown in gray because there positional but not sequence-based orthologs (see text and figure 5).

In all *Geobacter *species, acetate is the primary electron donor and it is oxidized via the TCA cycle, generating NADH, NADPH, and reduced ferredoxin (Figure 2) [7,23,24].

Acetate transporters were conserved in all the *Geobacter *species. There were two families of acetate transporters [25] that were conserved in all six species; one family contained a single ortholog from each species (GSU0518 in family 1057), and the second had multiple orthologs in each species (GSU1068, GSU1070, GSU2352 in 15) (see Additional file 6).

The genes for the eight reactions for acetate oxidation via the TCA cycle were conserved in all species (Figure 2). All of the subunits for acetyl-CoA transferase, citrate synthase, aconitase, isocitrate dehydrogenase, keto/oxoacid ferredoxin oxidoreductase, succinate dehydrogenase (complex II), fumarase, and malate dehydrogenase were conserved in all six of the species (see Additional file 6).

**Figure 3:**
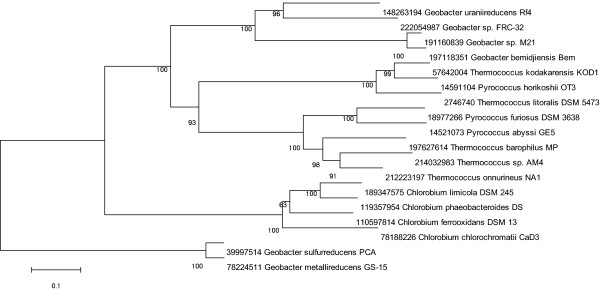
**Neighbor-joining phylogeny of the large subunit of the four-subunit hydrogenase**. This enzyme is specific to the *Geobacter *species of the subsurface clade, there are no orthologs in other *Geobacter *species. NCBI identification numbers of homologs and bootstrap values from 1000 replicates are shown.

*G. sulfurreducens, G. bemidjiensis, G. *strain FRC-32, and *G. lovleyi *can also use hydrogen as an electron donor in addition to acetate. In *G. sulfurreducens *the enzyme required for hydrogen oxidation has been identified as a four-subunit NiFe hydrogenase (GSU0782-GSU0785) [26]. This uptake hydrogenase was not conserved in all six of the *Geobacter *species; orthologs to the four subunits of this enzyme were found in three of the species that oxidize hydrogen: *G. sulfurreducens, G. bemidjiensis*, and *G. lovleyi*, and in one that does not: *G. uraniireducens *(see Additional file 6). No orthologs to this hydrogenase were found in *G. *strain FRC, which can oxidize hydrogen (see Additional file 6). However, *G. *strain FRC-32 and the other species isolated from the subsurface all encoded an additional hydrogenase, a four-subunit hydrogenase found only in these species (families 177, 178, 3032, and 3033, see Additional file 5). The four genes were similar to the heterotetrameric hydrogenase found in *Pyrococcus *that is a cytoplasmic, NADP-using hydrogenase [27] (Figure 3).

After acetate or hydrogen oxidation, the electrons are transferred into an inner-membrane bound electron transport chain, and protons are pumped out of the cytoplasm for ATP synthesis via an ATP synthase (Figure 2). *G. sulfurreducens *encodes two NADH dehydrogenases (complex I), one with 12 subunits and one with 14. This reaction is predicted to be the only one at which protons are pumped during Fe(III) or fumarate respiration [20]. All six *Geobacter *species contained orthologs to one of these enzymes, the 14-subunit enzyme (GSU0338-GSU0351) (see Additional file 6). The 12-subunit enzyme was conserved in all species except *G. loveyi *(see Additional file 6). The putative NADPH dehydrogenase [28] was conserved in all six *Geobacter *species (Figure 2, Additional file 6).

Regardless of whether acetate or hydrogen is the electron donor, ATP is synthesized with an inner-membrane bound ATP synthase. *G. sulfurreducens *encoded one ATP synthase enzyme in two gene clusters (GSU0108-GSU0114 and GSU0333-GSU0334). All six *Geobacter *species contained orthologs to all subunits of this enzyme (Figure 2, Additional file 6).

### The best conserved proteins in the *Geobacter *species

The level of sequence similarity among conserved proteins was estimated using bit score ratios between reciprocal orthologs [29]. 1266 *G. sulfurreducens *proteins had reciprocal orthologs in every other *Geobacter *genome (see Additional file 7). The average bit score ratio of these proteins was 70%. Only a small subset, 61 proteins, had an average bit score ratio of at least 90% (Table 3). This subset of very well conserved proteins contained housekeeping proteins, including ribosomal proteins, translation elongation factors, Rho transcription termination factor, and amino acid biosynthetic genes (Table 3). This set also included proteins involved in electron transfer, including subunits of the NADH dehydrogenase and the ATP synthase from the inner membrane, and the citrate synthase and acetyl-CoA transferase from the TCA cycle (Table 3). Other high-scoring proteins (at least 85%) included subunits of many of the other TCA cycle enzymes: succinate dehydrogenase, aconitase, malate dehydrogenase, 2-oxoglutarate oxidoreductase (see Additional file 7).

**Table 3:** Proteins with orthologs in every genome and average bit score ratio ≥ 90%

NCBI ID	*Geobacter sulfurreducens *gene	average score	Product Name
39995224	GSU0113	0.97	ATP synthase subunit B
39996849	GSU1750	0.96	translation initiation factor IF-1
39998183	GSU3093	0.96	ribosomal protein S21
39997962	GSU2871	0.96	translation elongation factor Tu
39996935	GSU1836	0.95	nitrogen regulatory protein P-II
39996567	GSU1467	0.95	iron-sulfur cluster-binding protein
39998198	GSU3108	0.94	transcription termination factor Rho
39996934	GSU1835	0.94	glutamine synthetase
39997943	GSU2851	0.94	ribosomal protein S3
39996069	GSU0966	0.94	hypothetical protein GSU0966
39996042	GSU0939	0.94	nitrogen regulatory protein
39997970	GSU2879	0.94	3-isopropylmalate dehydrogenase
39995222	GSU0111	0.94	ATP synthase subunit A
39998429	GSU3340	0.94	60 kDa chaperonin
39998543	GSU3454	0.93	radical SAM domain protein
39995273	GSU0162	0.93	aspartate aminotransferase
39996704	GSU1604	0.93	acyl carrier protein
39995448	GSU0339	0.93	NADH dehydrogenase I, B subunit
39996890	GSU1791	0.93	ATP-dependent protease
39995447	GSU0338	0.93	NADH dehydrogenase I, A subunit
39998155	GSU3064	0.93	cell division protein FtsA
39997187	GSU2089	0.93	rod shape-determining protein MreB
39997925	GSU2833	0.93	30S ribosomal protein S11
39997939	GSU2847	0.92	ribosomal protein L14
39995936	GSU0830	0.92	heavy metal efflux pump
39995264	GSU0153	0.92	argininosuccinate synthase
39997961	GSU2870	0.92	ribosomal protein L33
39995685	GSU0578	0.92	glycyl-tRNA synthetase
39998185	GSU3095	0.92	imidazoleglycerol phosphate synthase
39997958	GSU2867	0.92	ribosomal protein L11
39995210	GSU0099	0.92	MglA protein
39995598	GSU0490	0.92	acetyl-CoA hydrolase/transferase
39997634	GSU2539	0.92	saccharopine dehydrogenase
39997914	GSU2821	0.91	nitrogenase iron protein
39998542	GSU3453	0.91	uroporphyrinogen decarboxylase
39997117	GSU2019	0.91	acetyl-CoA carboxylase
39997929	GSU2837	0.91	preprotein translocase SecY
39995209	GSU0098	0.91	MglB protein
39995271	GSU0160	0.91	dihydrodipicolinate reductase
39995450	GSU0341	0.91	NADH dehydrogenase I, D subunit
39995452	GSU0343	0.91	NADH dehydrogenase I, F subunit
39998388	GSU3299	0.91	carboxyl transferase domain protein
39996631	GSU1531	0.90	phosphoribosyl-AMP cyclohydrolase
39996591	GSU1491	0.90	type IV pilus biogenesis protein PilB
39997632	GSU2537	0.90	arginine decarboxylase
39996434	GSU1332	0.90	heavy metal efflux pump
39998197	GSU3107	0.90	ribosomal protein L31
39997384	GSU2286	0.90	enolase
39996898	GSU1799	0.90	aspartate kinase
39997946	GSU2854	0.90	50S ribosomal protein L2
39995201	GSU0090	0.90	heterodisulfide reductase subunit
39998045	GSU2954	0.90	arsenical-resistance protein
39996208	GSU1106	0.90	citrate synthase
39996368	GSU1266	0.90	GTP-binding protein LepA
39995246	GSU0135	0.90	delta-aminolevulinic acid dehydratase
39998096	GSU3005	0.90	thiamine biosynthesis protein ThiC
39997945	GSU2853	0.90	ribosomal protein S19
39995451	GSU0342	0.90	NADH dehydrogenase I, E subunit
39997952	GSU2860	0.90	translation elongation factor G
39997010	GSU1912	0.90	dihydroxy-acid dehydratase
39998398	GSU3309	0.90	hypothetical protein GSU3309

### Identifying genes encoding cytochromes

After the electron donors have been oxidized and the electrons have been transferred into the inner membrane, they must then be transferred out of the cell to the extracellular electron acceptor like Fe(III) or electrodes. This pathway presumably requires periplasmic and outer-membrane *c*-type cytochromes, an abundance of which is the hallmark of *Geobacter *species [30,32-34]. Several cytochromes have been shown to be required for optimal growth by Fe(III) reduction or on electrodes in *G. sulfurreducens*: PpcA[12], MacA[13], OmcB[11], OmcE[16], OmcF[15], OmcG[35], OmcH[35], OmcS[16], OmcT[16], OmcX (M. Izallalan, unpublished), and OmcZ (B.C. Kim, unpublished).

Searching all six *Geobacter *species genomes showed that at least 100 ORFs in each genome contained at least one occurrence of the motif for covalent heme binding (CXXCH), indicating that these may be cytochromes (see Additional file 8). This was more than was found in 16 other genomes including those of *Shewanella, Desulfovibrio, Rhodoferax*, and *Anaeromyxobacter *species known to be cytochrome rich (see Additional file 8). Since this definition of cytochrome is minimal, a more stringent definition was created using 26 sequence profiles described in the protein database Interpro as *c*-type cytochromes. These profiles were compared against all of the proteins in the six *Geobacter *genomes. Proteins were considered cytochromes if their sequence contained at least one profile match and at least one CXXCH motif (see Additional file 9).

These results showed that each *Geobacter *genome contained an average of 79 cytochromes (Table 4). *G. uraniireducens *contained the most cytochromes, 104, and *G. lovleyi *the least, 61. On average, 2.1% of the proteins encoded in the genome of each of the *Geobacter *species are cytochromes (Table 4). Not only is the number of cytochromes in the genomes large, 85% of the cytochromes contain more than one heme motif - with 7.7 hemes per cytochrome on average (Table 4).

**Table 4:** Characteristics of cytochromes found in each genome

	**ORFs in genome**	**cytochromes total**	**cytochromes (% genome)**	**cytochromes with >1 heme**	**cytochromes (% multiheme)**	**hemes per cytochrome (average)**
	
*G. bemidjiensis*	4018	73	1.8	65	89.0	7.6
*G. lovleyi*	3685	61	1.7	46	75.4	4.8
*G. metallireducens*	3532	76	2.2	66	86.8	7.3
*G. strain FRC-32*	3396	68	2.0	58	85.3	9.6
*G. sulfurreducens*	3446	89	2.6	78	87.6	7.5
*G. uraniireducens*	4357	104	2.4	91	87.5	9.3
average	3739	79	2.1	67	85.3	7.7

### Conservation of cytochromes

While an abundance of cytochromes are found in all of the *Geobacter *species, very few were conserved in all six species, in contrast to the excellent conservation seen for the other energy metabolism proteins discussed above. There were 471 cytochromes in total identified in the six *Geobacter *genomes (see Additional file 9). Only 64 of the cytochromes (14%) were part of a protein family that included at least one cytochrome from each of the six genomes. These 64 conserved cytochromes formed nine protein families (Table 5).

**Table 5:** Characteristics of cytochrome families with members in every genome

ID	members	*Geobacter sulfurreducens *gene	CXXCH motifs	description
45	10	GSU1761	3	
49	9	GSU0364	3	ppcA and ppcB
51	9	GSU2732	8	orf2 OmcBC operon
71	8	GSU2937	5	bc complex
266	6	GSU2935	12	bc complex
267	6	GSU2934	10	bc complex
271	6	GSU2930	2	bc complex
1292	6	GSU0592	12	
1467	6	GSU0274	9	inner membrane

There was poor conservation across the species of many of the cytochromes that have been shown to be required *in vivo *in *G. sulfurreducens *for wild-type levels of Fe(III) or electrode reduction (Figure 2). Most of the cytochromes required in *G. sulfurreducens *for growth on extracellular acceptors were not conserved in all species, including OmcE, OmcF, OmcS, OmcT, OmcX, OmcZ, and MacA (Figure 2, Additional file 9).

Only one of the nine well-conserved cytochrome families contained a cytochrome, PpcA, known to be required for wild type levels of Fe(III) reduction [12]. At least one homolog to PpcA was found in every genome, and there were multiple homologs in most of the genomes: five in *G. sulfurreducens*, five in *G. metallireducens*, four in *G. uraniireducens*, three in *G. bemidjiensis*, two in *G. *strain FRC-32, and one in *G. lovleyi *(see Additional file 2). In related sulfate- and sulfur-reducing δ-*Proteobacteria *species, the most abundant and best studied cytochromes are the of the tetra-heme *c*_3 _type [36,37], while those of PpcA family are of the tri-heme *c*_7 _type [38].

**Figure 4:**
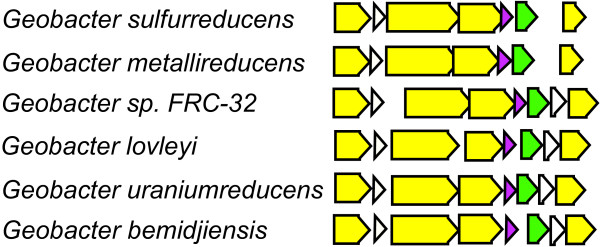
**The gene cluster (GSU2937 through GSU2930) encoding the putative inner-membrane cytochrome *bc *complex that is conserved in all six *Geobacter *species**. Genes encoding *c*-type cytochromes are shown in yellow, the Fe-S cluster protein encoding gene is shown in purple, and the cytochrome *b *gene is shown in green. All of these protein are orthologs across all of the *Geobacter *genomes (Table 5). The *c*-type cytochromes contain 2, 10, 12, and 5 heme-binding motifs each, respectively (see Additional file 9).

Analysis of the well-conserved cytochromes showed that four of the nine families conserved in all species were encoded together in a single cluster in each genome (Figure 4, Table 5). These conserved cytochromes were predicted to be 2-heme (GSU2930), 10-heme (GSU2934), 12-heme (GSU2935), and 5-heme (GSU 2937) (see Additional file 9). Also in this cluster were an inner-membrane-bound *b*-type cytochrome (GSU2932) and Rieske Fe-S protein (GSU2933) (Figure 4), which were clearly homologous to the core of the cytochrome *bc *complexes [39,40]. The *b*-type cytochrome and the Rieske protein were also conserved in all of the genomes (see Additional file 2). In other species, the cytochrome *bc *complex (complex III) catalyzes a key step in electron transport, that which provides the electrical link between the inner membrane and periplasm. However, the protein that provides this link in the *Geobacteraceae *has not been characterized, making this well-conserved cluster a good candidate for further analysis. This enzyme is especially important because it may be a second possible location of proton pumping in the cell, which would affect ATP yield during respiration, and may be different depending on the electron acceptor or on the redox potential of the cell. Typically, there is a single *c*-type cytochrome associated with this enzyme, which is a tetra-heme cytochrome in other δ-*Proteobacteria *[41], so the role of the multiple *c*-type cytochromes in this highly-conserved cluster is novel and warrants further investigation.

There were three other cytochrome families that were well conserved in all six genomes: families of 3-heme, 9-heme, and 12-heme cytochromes (Table 5). None of these cytochromes have been studied.

### Duplications of cytochrome genes

Twenty-eight of the 115 families that included cytochromes had more than one protein member per genome (see Additional file 9). In other words, they included paralogs, which may represent duplicated cytochrome genes. The largest cytochrome family had 12 members from 5 genomes (family 23, Additional file 9). Several families were made up of cytochromes from only a single genome, indicating recent duplication or triplication of the cytochrome since that species diverged (families 3111, 3250, 3413, and 3597).

Several cytochromes known to be required for wild type Fe(III) metabolism appeared to have been duplicated within single genomes. The OmcS family (64) had nine members, all 6-heme cytochromes, found in four of the *Geobacter *genomes (see Additional file 9). The *G. bemidjiensis *genome contained four OmcS proteins, *G. sulfurreducens *three, and one in both *G. *FRC-32 and *G. uraniireducens *(see Additional file 9). The OmcZ family (2307) contained four members from three genomes: *G. sulfurreducens *had two members (see Additional file 9). All six of the *Geobacter *genomes contained more than one PpcA-like protein (Table 5).

**Figure 5:**
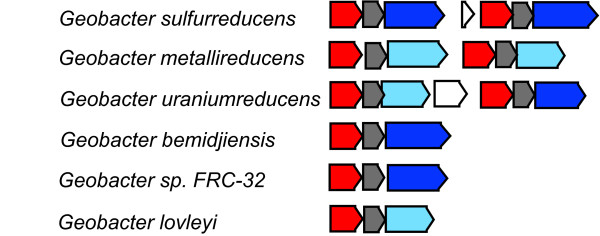
**The region of the operon of *omcB *(dark blue) in all six *Geobacter *species genomes**. In *G. sulfurreducens *the multi-heme cytochrome OmcB, which is required for electron transport to extracellular acceptors, is encoded in an operon with two other genes, *orf1 *(red) and *orf2*(gray) that is duplicated in the genome [54]. Shown here are regions of the genomes that encode the orthologs to these genes in all six *Geobacter *genomes, with orthologs colored identically. In some cases, there were multi-heme cytochromes encoded in the position of OmcB, but the sequence similarity was too low to confidently predict orthology, so these genes are colored light blue.

The Orf2 cytochromes (GSU2732 and GSU2738) were conserved across all of the *Geobacter *species (Table 5), and also showed duplication. There are nine members in this family (51), all 8-heme or 9-heme cytochromes (see Additional file 9). *G. sulfurreducens*, *G. metallireducens*, and *G. uraniireducens *each contain two Orf2 cytochromes. In *G. sulfurreducens*, the Orf2 genes are encoded in a tandem repeat with another duplicated cytochrome (called OmcB/OmcC) known to be important for Fe(III) reduction[11] (Figure 5). Initial examination of the OmcB/OmcC family (number 1653) indicated it did not have complete conservation like the Orf2 family did, but analysis of these genes in genome context indicates that an operon of similar structure was conserved in all six species (Figure 5). Alignment of the *orf2*-*omcB *genome regions from all six species showed that there was at least one operon with similarity to the *orf2-omcB *operon in each genome, and furthermore, there were tandem repeats of this operon in several of the genomes (Figure 5). Interestingly, while the Orf2 cytochrome gene and the Orf1 gene immediately upstream were well conserved orthologs across all species, the gene immediately downstream varied. In all cases there was a multi-heme cytochrome encoded in the OmcB/C spot in the operon, but the sequence similarity to OmcB/C varied (Figure 5). This indicates that this operon may be important in all six species, though while it appears that conservation of the sequence of the Orf2 cytochromes is important, there may be less pressure for the larger outer membrane cytochromes to maintain a specific sequence.

### Lateral gene transfer

The data presented above indicates that cytochromes are abundant in each genome, but not very well conserved across the genomes. Cytochrome duplication and divergence appears to have played a role in these genotypes. In addition, to investigate whether cytochromes were less well conserved because they were acquired laterally rather than inherited vertically, genes originating from lateral gene transfer were identified using a combination of phylogenetic and BLAST-based analysis. A neighbor-joining phylogenetic tree was inferred for every protein from the six genomes and homologous sequences for each protein were selected from the non-redundant protein database. These trees were used to identify proteins for which the nearest relative was not from the *Geobacteraceae*. If the phylogeny was strongly supported (bootstrap ≥ 50) or if the phylogeny was weakly supported and the most similar sequence in the non-redundant protein was not a *Geobacteraceae *species, the protein was considered a lateral gene transfer candidate.

2,196 of the 21,434 proteins in these six genomes (9.8%) were predicted to have originated from recent transfer from a distantly related organism (see Additional file 1). Only 19 of the 472 predicted cytochromes (4.0%) were identified as lateral gene transfer candidates - 1 in *G. bemidjiensis*, 5 in *G. lovleyi*, 6 in *G. metallireducens*, 2 in *G. sulfurreducens*, and 3 in *G. uraniireducens *(see Additional file 9). None of the cytochromes shown to be required for wild type electron transport in *G. sulfurreducens *were predicted to have originated from lateral gene transfer (see Additional file 9). These data indicated that the abundance of cytochromes in these six species cannot be explained by frequent lateral gene transfer.

## Conclusions

The results show that the genes for oxidizing acetate and transferring electrons to cytoplasmic carriers, and for inner membrane electron transport, are well conserved between the *Geobacter *genomes. These results indicate that the *Geobacter *species and their last common ancestor all oxidized acetate using the same TCA cycle pathway that produces NADH, NADPH, and reduced ferredoxin. These substances are then oxidized at the inner membrane, and ATP is generated via oxidative phosphorylation. The previously unidentified site of quinol oxidation in the inner membrane is suggested to be a cytochrome *bc *complex encoded in an unique gene cluster that is conserved in all six species. The pathways used by the better-studied species were also found to be conserved in the newly discovered species that predominate in subsurface environments undergoing bioremediation, suggesting that the current metabolic model for *G. sulfurreducens*[20] provides a good foundation for broader modeling of microbial metabolism in contaminated subsurfaces during bioremediation. However, the role of the newly identified hydrogenase unique to these subsurface species merits further investigation.

In stark contrast to the conservation of the pathway for ATP generation from acetate is the lack of conservation of the enzymes that dispose of the electrons after ATP production. The six *Geobacter *genomes contain an average of 79 cytochrome genes each, with each cytochrome predicted to bind an average of more than 7 hemes. So an abundance of extracytoplasmic heme is clearly important in these species. However, only 14% of the cytochromes are conserved in all six of the genomes. More surprisingly, even the cytochromes that have been shown to be required in *G. sulfurreducens *for electron transport to Fe(III) or electrodes are not well conserved.

Cells of *G. sulfurreducens *have been shown to be capable of storing ca. 1.6 × 10^-17 ^mol electrons in the iron of their cytochromes [42]. This has lead to the proposal that cytochromes may act as electric capacitors, accepting and storing the electrons from energy metabolism for short time spans in the absence of an extracellular electron-accepting surface [43]. The data presented here indicates that in these species there is a combination of strong pressure to maintain many cytochrome genes with weak pressure to maintain the sequence of most cytochrome genes. This lack of conservation of cytochrome genes suggests that in *Geobacter *species there may not be a single common pathway for electron transport outside the cell, and that cytochromes may be required for general Fe-bearing capacity, as sinks for electrons between the inner-membrane electron transport chain and the extracellular acceptor.

## Methods

### Genome sequencing and annotation

With the exception of *G. sulfurreducens *[5], sequence data for the genomes were produced by the US Department of Energy Joint Genome Institute http://www.jgi.doe.gov, using a whole-genome shotgun strategy for the Sanger-sequencing of 3-Kb, 8-Kb, and 40-Kb DNA libraries to 8-9X depth. Open reading frames and their translations and predicted function based on automated annotation were taken from NCBI http://www.ncbi.nlm.nih.gov/, and are listed in Additional file 1 of the supplementary material. The following motifs were used to annotate cytochromes (showing Interpro identification http://www.ebi.ac.uk/interpro/): IPR000298, IPR000763, IPR000883, IPR000883, IPR001128, IPR002016, IPR002016, IPR002321, IPR002322, IPR002585, IPR003317, IPR004203, IPR009056, IPR010176, IPR010177, IPR010255, IPR010960, IPR011031, IPR011048, IPR012282, IPR012292, SSF47175, SSF48613, SSF48695, SSF81342, SSF81648.

### Clustering orthologs into protein families

All proteins in the genomes were clustered into families of orthologs and recent paralogs using OrthoMCL [19], which uses reciprocal best similarity pairs from all-vs-all BLAST [44] to identify orthologs and recent paralogs, which are then clustered together across all the genomes using the Markov clustering algorithm [45]. A functional role was predicted for each cluster using the *G. sulfurreducens in silico *model annotation [20] and COG categorization [21]. The level of sequence similarity among conserved proteins was estimated using bit score ratios between reciprocal orthologs [29].

### Phylogenetics

All the ORFs from the six genomes and the outgroup species *Pelobacter propionicus *(NC_008609) were put into orthologous groups using Hal [46], with inflation parameters from 1.1-5.0 for the clustering algorithm. The proteins used for the phylogeny were those that were part of a cluster generated with any inflation value that had exactly one member from each genome, and are listed in Additional file 4 of the supplementary material. All of the proteins in the cluster were concatenated and the resulting sequences aligned by ClustalW [47]. ProtTest [48] was used to select a model of molecular evolution and MrBayes [49] was used to create a Bayesian estimation of the phylogeny. The single gene phylogeny was inferred from a ClustalW[47] alignment of homologs to the large subunit of the hydrogenase from the NCBI non-redundant database. Distances and branching order were determined by the neighbor-joining method[50] with bootstrap values from 1000 replicates in Mega[51].

### Lateral gene transfer

A phylogenetic tree was inferred using PhyloGenie [52] for every protein from the six genomes. Homologous sequences for each protein were selected by BLAST from the non-redundant protein database from NCBI http://www.ncbi.nlm.nih.gov/, alignments were created with ClustalW [47], and the phylogeny was inferred using neighbor-joining [50] and 100 bootstrapped replicates. If, for a given protein, a phylogenetic relationship with non-*Geobacteraceae *was strongly-supported (bootstrap ≤ 50) or if the relationship was weakly supported and the most similar sequence in the non-redundant protein database from NCBI was not a *Geobacteraceae *species, the protein was considered a candidate. If the next branch out contained a single sequence not from *Geobacteraceae *species, the query gene was defined as being from lateral transfer. If the next branch contained a single sequence from *Geobacteraceae*, it was not. If the sister group was a clade or was not strongly supported, the ancestral condition was inferred [53] and used to determine lateral transfer.

## Authors' contributions

JEB carried out the conservation analysis, created the physiological models, constructed the single gene phylogenies, and drafted the manuscript. NDY designed the method for and carried out the lateral gene transfer prediction, constructed the whole genome phylogeny, and carried out the clustering method. DRL conceived of the study and helped draft the manuscript. All authors read and approved the final manuscript.

## Supplementary Material

Additional file 1 **All proteins referenced in this study**. Spreadsheet with NCBI identification numbers and descriptions including name, predicted function, COG membership, protein family ID, family conservation pattern, and lateral transfer prediction. Click here for file

Additional file 2 **Protein families and their members from each of the genomes**. Spreadsheet showing protein families of orthologs, with descriptions including ID, predicted function, member proteins, member genomes, family size, and conservation pattern. Click here for file

Additional file 3 **Frequency of the phyletic patterns of protein conservation**. Spreadsheet showing the number of proteins with a given pattern of conservation. Click here for file

Additional file 4 **Proteins used in the whole genome phylogeny**. Spreadsheet showing the IDs of the proteins used. Click here for file

Additional file 5 **Proteins conserved only within the species in the subsurface clade**. Spreadsheet showing the proteins that were conserved in all and only the species in the subsurface clade including ID, protein family ID, conservation of family, and predicted function. Click here for file

Additional file 6 **Conservation of proteins involved in the energy metabolism of anaerobic respiration **Spreadsheet showing all of the proteins with their metabolic role, conservation pattern, reaction abbreviation in the constraint-based model, protein family, and genomes of family members. Click here for file

Additional file 7 **Proteins conserved in all genomes (with reciprocal orthologs in every genome)**. Spreadsheet showing all of the proteins that had reciprocal best BLAST matches in every single other genomes with their metabolic role, reaction abbreviation in the constraint-based model, bit score ratio for the reciprocal best BLAST match in every other genome, and average bit score ratio. Click here for file

Additional file 8 **Total heme motifs in 23 cytochrome-rich genomes**. Spreadsheet showing totals of heme binding motifs (CxxCH) in 23 completed genomes, including total genes with heme motif(s), most hemes per gene, number of genes with more than one motif and percent with more than one motif. Click here for file

Additional file 9 **Cytochromes in all *Geobacter *genomes**. Spreadsheet showing all of the proteins predicted to encode cytochromes in all of the *Geobacter *genomes, with the number of heme binding motifs, protein family, conservation pattern, number of members in the family, lateral transfer prediction, published gene name, and paralog prediction. Click here for file
